# Aberrant amplitude of low-frequency fluctuations in different frequency bands and changes after one-night positive airway pressure treatment in severe obstructive sleep apnea

**DOI:** 10.3389/fneur.2022.985321

**Published:** 2022-08-22

**Authors:** Yuanfeng Sun, Sophine Xin Yang, Min Xie, Ke Zou, Xiangdong Tang

**Affiliations:** ^1^Sleep Medicine Center, West China Hospital, Sichuan University, Chengdu, China; ^2^Business Administration of Business School, Sichuan University, Chengdu, China; ^3^Mental Health Center, West China Hospital, Sichuan University, Chengdu, China

**Keywords:** obstructive sleep apnea, CPAP, frequency band, restingstate functional magnetic resonance imaging, amplitude of low-frequency fluctuation (ALFF), brain function

## Abstract

**Objective:**

This study was aimed to investigate the characteristics of the amplitude of low-frequency fluctuation (ALFF) at specific frequencies in severe obstructive sleep apnea (OSA) patients. A comparison was made between pre-CPAP treatment and one night after continuous positive airway pressure (CPAP) treatment.

**Methods:**

30 severe OSA patients and 19 healthy controls (HC) were recruited. The ALFF method was used to assess the local features of spontaneous brain activity and calculated at different bands (slow-5 and slow-4). A correlation analysis was performed to evaluate the relationship between the changes of the ALFF and polysomnography data.

**Results:**

Compared with HC, in slow-5 frequency band, OSA patients showed significantly decreased ALFF in the left inferior temporal gyrus, and significantly increased ALFF in the left middle frontal gyrus, left inferior frontal gyrus, triangular part, right superior frontal gyrus, dorsolateral and right middle temporal gyrus. In slow-4 frequency, there was significantly decreased ALFF in the right inferior temporal gyrus, and significantly increased ALFF in the left precuneus, right posterior cingulate gyrus and right median cingulate besides the slow-5 difference band showed. Compared with pre-CPAP, we found that after CPAP treatment, ALFF signals in the left insula in slow-5 and left caudate in slow-4 increased, but the calcarine in slow-4 significantly reduced. Correlation analysis showed that the left angular slow-4 band change was positively correlated with the slow wave sleep change (*r* = 0.4933, *p* = 0.0056). The left cerebellum 6 slow-5 band change was positively correlated with the duration of the REM sleep change (*r* = 0.4563, *p* = 0.0113), and the left cerebellum 6 slow-4 band change was also positively correlated with the mean blood oxygen change in the REM (*r* = 0.4591, *p* = 0.0107) and NREM sleep (*r* = 0.4492, *p* = 0.0128).

**Conclusion:**

We found that the use of slow-4 was more specific in OSA studies. These results suggested that the severe OSA patients have frequency-related abnormal spontaneous neural activity, which may contribute to a better understanding of the pathological basis of OSA-related diseases and provide a potential therapeutic target for OSA patients.

## Introduction

Obstructive sleep apnea syndrome (OSA) is a common sleep disorder characterized by frequent upper airway collapse during sleep, leading to intermittent hypoxemia, sleep fragmentation and changes in sleep structure (1, 2). OSA is associated with a range of harmful effects, including excessive daytime sleepiness, increased risk of work-related injuries and reduced quality of life. In addition, OSA is a multisystem chronic disease which often comorbid to various other diseases, such as heart disease, hypertension, cerebrovascular disease, reflux esophagitis, depression, anxiety, insomnia, etc. (3–6). To date, the underlying pathophysiological mechanisms associated with OSA are not fully understood.

Non-invasive neuroimaging techniques have been widely used to explore the pathophysiological mechanisms of various diseases, such as neurological and psychiatric disorders. Different neuroimaging techniques have been used to explore related structural, functional, and metabolic alterations in OSA patients (7–9). Many previous studies (10–13) using structural neuroimaging have suggested that OSA patients may have brain tissue damage. Resting-state functional magnetic resonance imaging (rs-fMRI) is an oxygen-dependent brain functional imaging method, which can give more accurate structural and functional relationships (14). As a reliable measure of rs-fMRI techniques, the amplitude of low-frequency fluctuations (ALFF) has been widely applied to assess spontaneous neural activity related to brain metabolism and brain function (15). Therefore, using rs-fMRI techniques such as ALFF measurement to conduct in-depth research on OSA patients and explore their brain changes has profound significance.

To date, most rs-fMRI studies have detected spontaneous low-frequency oscillatory (LFO) activity in a specific frequency band of 0.01–0.08 Hz. It is proposed that neural oscillations with different frequencies in the human brain may be sensitive to activity in different regions and can be used to reflect different physiological functions of brain activity (16). The rs-fMRI LFO can be divided into four different frequency bands [slow-5 (0.01–0.027 Hz), slow-4 (0.027–0.073 Hz), slow-3 (0.073–0.198 Hz) and slow-2 (0.198–0.25 Hz)] (17, 18). However, only the traditional 0.01–0.08 Hz spectrum has been used to investigate OSA patients (19, 20). These researches (19, 20) reported that OSA patients had higher ALFF values in the right middle cingulate, left medial superior frontal gyrus, right anterior cingulate, right hippocampus, and right inferior occipital gyrus. The ALFF values were lower in the left inferior temporal gyrus and right cerebellum. In our study, the slow-5 and slow-4 bands were examined because they contain most of the traditional 0.01–0.08 Hz spectrum and there is minimal overlap with potential physiological noise frequencies. For example, Hoptman's study (21) showed that patients with schizophrenia were generally abnormal in the slow-4 band. Wang's study (22) also suggested that the brain's intrinsic activity patterns were sensitive to specific frequency bands such as slow-4 and slow-5 in Parkinson's disease patients. To our knowledge, no research has investigated the frequency-dependent ALFF in OSA patients. It would be necessary to differentiate the frequency bands to examine the ALFF in OSA patients.

Continuous positive airway pressure (CPAP) is the gold standard treatment for moderate/severe OSA patients (23). Previous studies have shown that brain damage may occur in OSA patients due to long-term sleep fragmentation and hypoxia, which can be ameliorated by CPAP. For example, brain structural imaging studies showed that after 3 months of CPAP treatment, partial white matter integrity was restored (24, 25), as well as the moderate improvements in mood, attention, and executive function. In addition, OSA patients treated for a longer period (6 or 12 months) had brain metabolite levels and brain volume changes in gray (26) and white matter integrity (24) in multiple regions of the brain which were similar to those of well-sleep people, suggesting that chronic brain damage in OSA patients can be reversed by CPAP treatment.

However, after one-night CPAP treatment, the proportion of slow wave sleep and REM sleep in severe OSA patients increased, the arouse index and apnea-hypopnea index (AHI) decreased significantly, and the blood oxygen saturation improved significantly. Furthermore, the next day, most patients' daytime sleepiness was alleviated and their attention and responsiveness improved significantly (27). However, there is no report on brain function related to such significant changes after one-night CPAP treatment in severe OSA patients. Therefore, this study hypothesized that the brain regions of OSA patients are also altered after one-night CPAP treatment.

## Method

### Subjects

Thirty newly diagnosed, untreated male OSA patients and nineteen healthy controls (HC) were recruited for this study from the Sleep Medicine Center of West China Hospital, Sichuan University. In this study, all subjects were recruited based on the following criteria: (1) male, (2) OSA patients with AHI>30, HC AHI <5, (3) age 20-60 years. In addition, the following characteristics were excluded: (1) non-right-handed. (2) A history of neurological or psychiatric diseases (such as neurodegenerative diseases, epilepsy, brain trauma, depression, bipolar disorder) or other sleep disorders (such as insomnia); (3) MRI contraindications (such as metal implants); (4) shift work; (5) abuse of illicit drugs. All subjects underwent Epworth sleepiness scale (ESS) assessments before undergoing polysomnography (PSG). All subjects signed written informed consent forms for this study before data acquisition and the study protocol was approved by the Human Research Ethics Committee of West China Hospital, Sichuan University.

### Polysomnography

All subjects were required to undergo PSG evaluation (Alice 6, Respironics, Orlando, FL, USA). According to the American Academy of Sleep Medicine guidelines, electroencephalography (EEG), electromyogram (EMG), electrooculography (EOG), electrocardiogram (ECG), oral and nasal airflow, snoring, thoracic and abdomen breathing movement, oxygen saturation (SaO_2_) and body position were recorded. The subjects' tests were recorded by infrared cameras and continuously monitored by an experienced sleep technologist.

### Continuous positive airway pressure (CPAP) treatment

On the following day, all OSA patients received CPAP treatment for one night (22:30-−6:30) after PSG evaluation. The treatment pressure of the ventilator was set at 4–20 cm H_2_O, and the treatment pressure was set automatically.

### fMRI data acquisition

All participants had functional and structural MRI images collected in our hospital using a 3.0 T MRI scanner (Siemens, Trio, Germany). MRI scans were performed the following day at 7:30–8:30 am after PSG or CPAP monitoring, so the patients underwent two scans and the controls only had one scan. Rs-fMRI data were acquired using an echo planar imaging (EPI) sequence with the following parameters: repetition time (TR) = 2,000 ms, echo time (TE) = 30 ms, flip angle = 90°, thickness = 5.0 mm, gap = 0.5 mm, field of view (FOV) = 240 × 240, matrix size = 64 × 64, slices = 30; a total of 6,000 rs-fMRI images were recorded. High-resolution T1-weighted MRI images of brain structures were obtained using a sagittal three-dimensional T1-weighted magnetization prepared rapid acquisition gradient echo (MPRAGR) sequence: TR = 1,900 ms, TE = 2.34 ms, inversion recovery Time (TI) = 900 ms, flip angle = 90°, thickness = 1.0 mm, gap = 0.5 mm, FOV = 256 × 256, matrix = 256 × 256, layers = 176.

### Data preprocessing

We also evaluated the fMRI data for imaging or head motion-related artifacts before data preprocessing. Prior to ALFF analysis, DPARSFA (http://rfmri.org/DPARSF) and SPM12 (https://www.fil.ion.ucl.ac.uk/) based on MATLAB2018b (Math Works, Natick, MA, USA) were used to preprocess the fMRI data: (1) File format conversion from DICOM to NIFTI; (2) removal of the first 10 volumes; (3) slice timing and head motion correction; (4) T1 segmentation with the Diffeomorphic Anatomical Registration Through Exponentiated Lie algebra (DARTEL) spatial normalization into the Montreal Neurological Institute (MNI); (5) regression of nuisance covariates including linear trend, white matter signals, cerebral spinal fluid signal, and Friston-24 parameters of head motions; (6) smoothing with a 6-mm full width at half maximum Gaussian kernel.

### ALFF analysis

The mean square root of each voxel power spectrum was calculated as ALFF [slow-5 band (0.01–0.027 Hz), and slow-4 band (0.027–0.073 Hz)]. All ALFF maps were converted to z-maps by subtracting the global mean and dividing by the standard deviation. Subsequent statistical and correlation analyses were based on normalized ALFF plots.

### Statistical analysis

Data were assessed for normality (Shapiro–Wilk test), and Student *t*-tests were performed to assess between-group differences on demographical and sleep data that were normally distributed. Mann–Whitney *U*-test was performed to assess between-group differences in variables that were not normally distributed.

Whole-brain ALFF (slow-5 and slow-4) were compared between the OSA group and the control group using an independent sample *t*-test. Age, education level and head motion were imported as covariates. Whole-brain ALFF before and after CPAP treatment (slow-5 and slow-4) were tested using paired samples *t*-test. Head motion was imported as a covariate.

Whole-brain ALFF before and after CPAP treatment were correlated with sleep-related data, respectively, and regions with significant correlation results were selected as regions of interest (ROIs) and extracted (the results were presented in the Supplementary material). Then, we performed the changes between the mean ALFF values of brain regions before and after CPAP treatment in these ROIs and the changes between the sleep data before and after CPAP treatment for correlation analysis.

## Results

### Clinical characteristics

The demographic and polysomnography data are shown in Table 1. There were no significant differences in age, education, total sleep time (TST), and the time of REM sleep between the OSA patients and the HC groups. The OSA patients showed significantly higher Body Mass Index (BMI), ESS, AHI and the time of NREM sleep, but lower mean in SaO_2_ (blood oxygen saturation) of REM sleep, NREM sleep and TST, slow wave sleep (SWS) compared with HC. After one-night CPAP treatment, the time of REM sleep, SWS, the mean SaO_2_ of REM sleep, NREM sleep and TST increased significantly. AHI and the time of NREM sleep decreased significantly.

**Table 1:** Demographic and polysomnographic data of participants.

	**Pre-CPAP (*n* = 30)**	**CPAP night (*n* = 30)**	**Health control (*n* = 19)**	**P^a^**	**P^b^**
Age (years)	42.50 ±5.82	42.50 ±5.82	40.32 ±2.89	0.136	ns
BMI (kg/m^2^)	29.41 ±3.08	29.41 ±3.08	23.97 ±2.57	<0.001	ns
Education (years) §	13.63 ±2.36	13.63 ±2.36	14.37 ±4.23	0.566	ns
ESS	15.37 ±5.96	15.37 ±5.96	3.42 ±1.50	<0.001	ns
TST (min)	452.68 ±67.37	429.42 ±51.79	418.35 ±55.217	0.071	0.142
REM (min)	63.93 ±33.57	111.00 ±36.18	73.37 ±22.96	0.290	<0.001
NREM (min)	388.75 ±64.93	318.42 ±37.74	344.98 ±45.03	0.014	<0.001
SWS (min)§	6.10 ±13.70	32.22 ±42.15	16.24 ±16.90	0.004	<0.001
AHI (events/h)	71.01 ±21.08	13.56 ±8.65	3.50 ±1.633	<0.001	<0.001
REM SaO_2_ (%)	80.43 ±10.31	94.70 ±1.43	94.42 ±2.48	<0.001	<0.001
NREM SaO_2_ (%)	88.17 ±4.80	94.03 ±1.67	94.37 ±1.74	<0.001	<0.001
TOTAL SaO_2_ (%)	87.53 ±4.97	94.33 ±1.45	94.47 ±1.65	<0.001	<0.001

§* Non-parametric test (Mann–Whitney U). P^a^, test between pre-CPAP OSA patients and HC; P^b^, test between CPAP night and pr-CPAP OSA patients. REM, the time of REM stage sleep; NREM, the time of NREM stage sleep; SWS, slow wave time; ESS, Epworth sleepiness scale; TST, total sleep time, ns, non-statistically significant*.

### ALFF changes in specific frequency band

**Figure 1:**
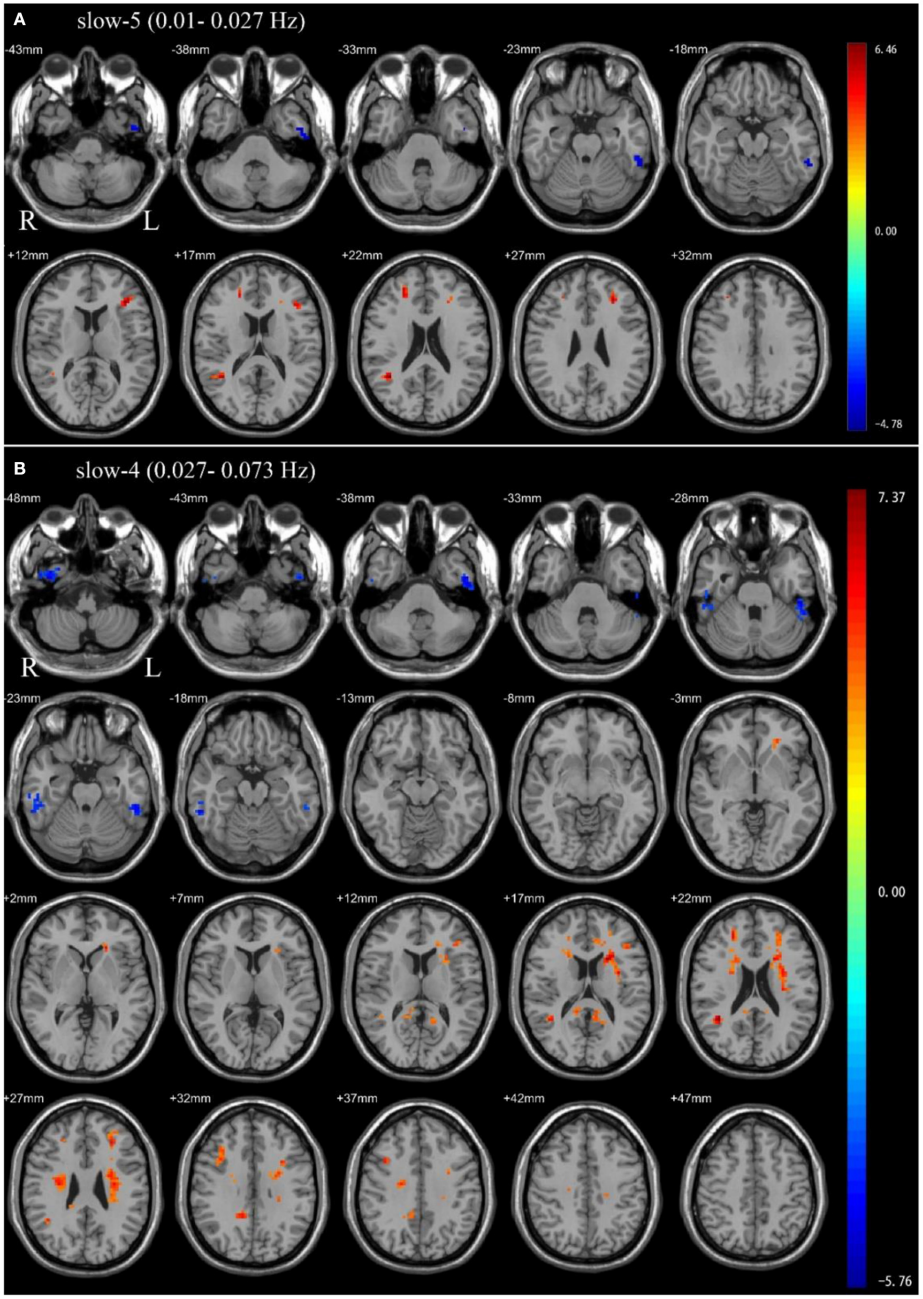
Independent sample *t*-test between health control groups and pre-CPAP OSA patients: **(A)** slow-5 frequency band (0.01–0.027 Hz). **(B)** slow-4 frequency band (0.027–0.073 Hz). Results were reported at voxel-level *p* < 0.001 and cluster-level *p* < 0.05, GRF corrected.

In the slow-5 frequency band, OSA patients showed significantly decreased ALFF in the left inferior temporal gyrus, and significantly increased ALFF in the left middle frontal gyrus, left inferior frontal gyrus, triangular part, right superior frontal gyrus, dorsolateral, right middle temporal gyrus compared to HC (Table 2 and Figure 1).

**Table 2:** The differences of ALFF in each frequency band between OSA groups and HC groups.

**Condition**	**Region**	**Brodmann area**	**Cluster size (voxels)**	**Cluster size (mm^3^)**	**MNI coordinates**			**Peak intensity:**
					**X**	**Y**	**Z**	
	**(A) Slow-5 frequency band (0.01–0.027 Hz)**							
OSA < HC	Temporal_Inf_L	BA20_L	31	837	−60	−42	0	−4.78389
	Temporal_Inf_L	BA20_L	26	702	−51	−12	−36	−4.70627
OSA>HC	Frontal_Inf_Tri_L	BA48_L	28	756	−39	30	15	6.46229
	Frontal_Mid_L	BA48_L	23	621	−27	33	27	5.80003
	Frontal_Sup_R	BA46_R	23	621	24	45	21	5.2604
	Temporal_Mid_R	BA39_R	24	648	39	−51	21	6.30294
	**(B) Slow−4 frequency band (0.027–0.073 Hz)**							
OSA < HC	Temporal_Inf_L	BA20_L	57	1,539	−51	−33	−27	−4.87198
	Temporal_Inf_L	BA20_L	31	837	−48	−6	−39	−4.93815
	Temporal_Inf_R	BA20_R	54	1,458	60	−42	−18	−4.80968
	Temporal_Inf_R	BA20_R	40	1,080	42	−6	−45	−4.74978
OSA>HC	Precuneus_L	BA29_L	29	783	−9	−48	15	5.06875
	Cingulum_Post_R	BA29_R	20	540	9	−39	15	4.73092
	Cingulum_Mid_R	BA23_R,	25	675	12	−45	33	5.27274
	Temporal_Mid_R	BA39_R	39	1,053	39	−51	21	7.13999
	Frontal_Inf_Oper_R	BA44_R	21	567	39	12	36	5.2112
	Frontal_Mid_L	BA46_L	40	1,080	−27	36	24	6.85861
	Frontal_Inf_Tri_L	BA48_L	20	540	−39	30	15	5.68692

*All clusters were reported with a voxel-level threshold of P < 0.001, GRF correction, and cluster-level of P < 0.05, two tailed*.

In the slow-4 frequency band, compared to HC, OSA patients showed significantly decreased ALFF in the bilateral inferior temporal gyrus, and significantly increased ALFF in the left precuneus, right posterior cingulate gyrus, right median cingulate, right middle temporal gyrus, right inferior frontal gyrus, opercular part, left middle frontal gyrus, left inferior frontal gyrus, triangular part (Table 2 and Figure 1).

### ALFF differences between pre-CPAP and post-CPAP OSA patients

**Figure 2:**
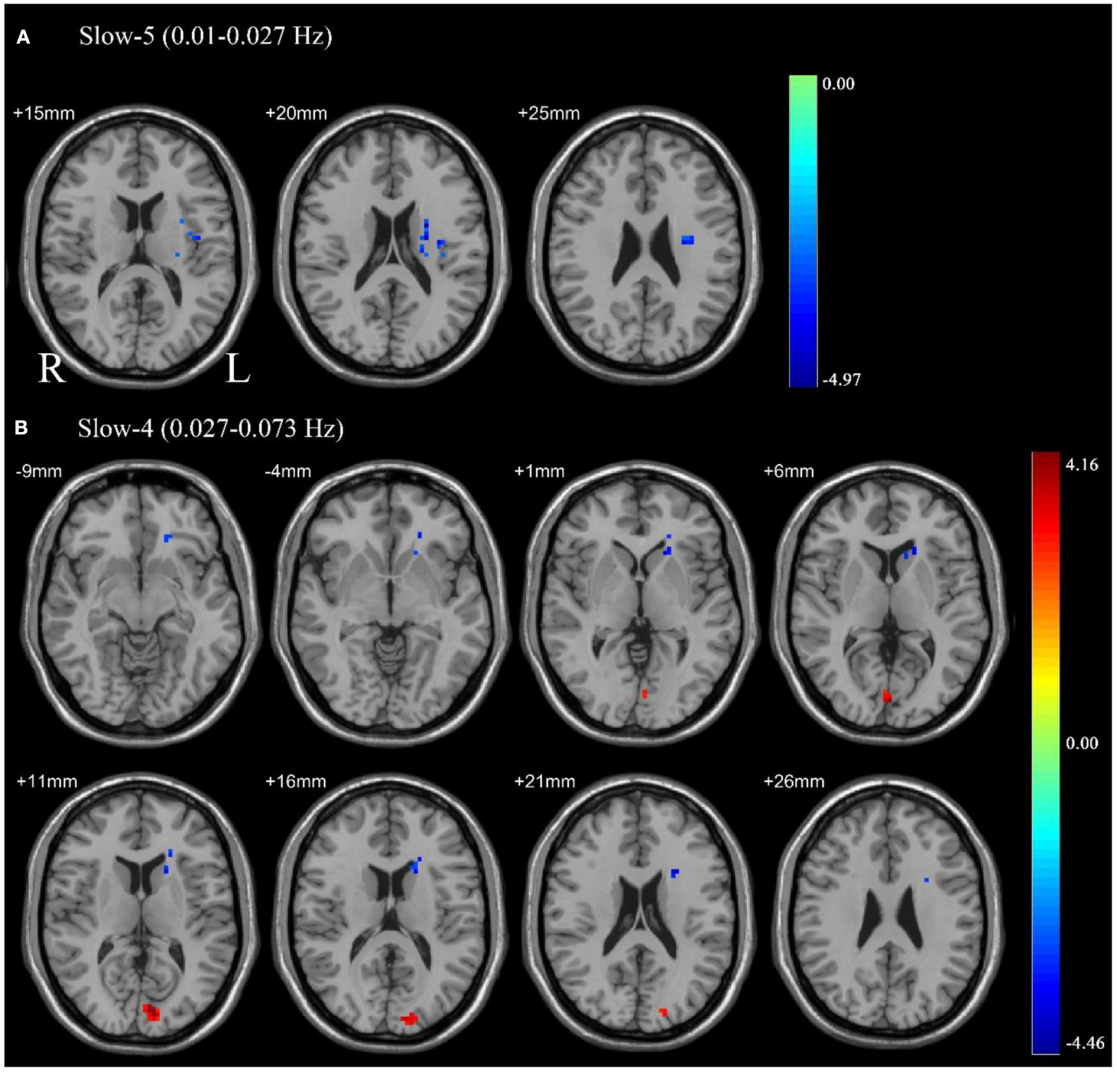
Pair sample *t*-test in the slow-5 frequency band (0.01–0.027 Hz) **(A)**, the slow-4 frequency band (0.027–0.073 Hz) **(B)**. All results were reported at voxel-level *p* < 0.01 and cluster-level *p* < 0.05, GRF corrected.

Paired sample *t*-tests were used to compare the difference in ALFF between pre-CPAP and post-CPAP OSA patients in the slow-4 and slow-5 frequency bands. Figure 2 showed significant differences of ALFF within different frequency bands. The detailed abnormal brain regions are shown in Table 3. We found that after CPAP treatment, ALFF signals in the left insula in the slow-5 and left caudate in the slow-4 increased, but the calcarine in the slow-4 significantly reduced.

**Table 3:** The differences of ALFF in each frequency band between the pre-CPAP and post-CPAP group.

	**Region**	**Brodmann area**	**Cluster Size (voxels)**	**Cluster Size (mm^3^)**	**MNI coordinates**			**Peak intensity:**
**Condition**					**X**	**Y**	**Z**	
	**(A) Slow-5 frequency band (0.01–0.027 Hz)**							
Pre-CPAP < post-CPAP	Insula_L	BA48_L,	53	1,431	−24	−21	18	−4.60315
	**(B) Slow-4 frequency band (0.027–0.073 Hz)**							
Pre-CPAP < post-CPAP	Caudate_L,	BA11_L	52	1,404	−16	16	18	−4.45842
Pre-CPAP> post-CPAP	Calcarine_L	BA18_L	43	1,161	0	−87	6	4.15529

*All clusters were reported with a voxel-level threshold of P <0.01, GRF correction, and cluster-level of P <0.05, two tailed*.

### Correlation analysis

**Figure 3:**
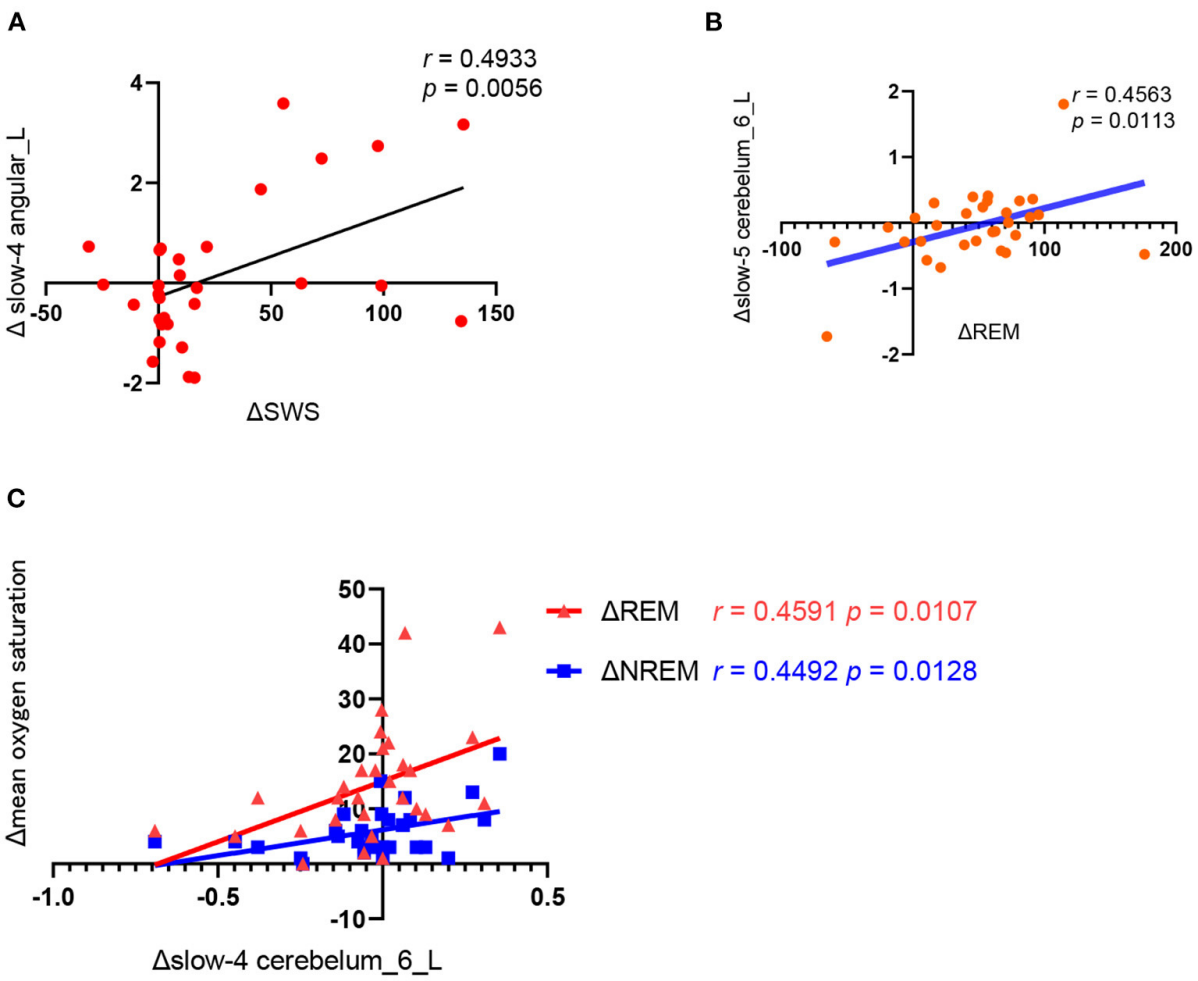
Correlation analysis between the changes in polysomnography parameters and ALFF values before and after CPAP titration. **(A)** ΔSWS (the SWS time of the CPAP night minus baseline); Δ slow4 angular_L(the slow-4 ALFF in the angular_L of post-CPAP minus the pre-CPAP), **(B)** ΔREM (the REM time of the CPAP night minus baseline), Δslow-5 cerebelum_6_L (the slow-5 ALFF in the cerebelum_6_L of post-CPAP minus the pre-CPAP). **(C)**.Δslow-4 cerebelum_6_L (the slow-4 ALFF in the cerebelum_6_L of post-CPAP minus the pre-CPAP), Δmean oxygen saturation (the mean oxygen saturation of REM or NREM of the CPAP night minus baseline).

As shown in Figure 3, the change of ALFF in the left angular of the slow-4 frequency band was positively correlated with the change of slow wave sleep duration (*r* = 0.4933, *p* = 0.0056) and the change of ALFF in left cerebellum 6 of the slow-5 frequency was positively correlated with the change of REM duration (*r* = 0.4563, *p* = 0.0113) before and after CPAP treatment. Moreover, the change of ALFF in left cerebellum 6 of the slow-4 frequency band was also positively correlated with the change of mean oxygen saturation during REM (*r* = 0.459, *p* = 0.0107) and NREM sleep (*r* = 0.4492, *p* = 0.0128).

## Discussion

For the first time, we used the slow-4 and slow-5 frequency bands of ALFF to analyze the characteristics of resting-state frequency in OSA patients and explored the changes of ALFF values in OSA patients after one-night CPAP treatment. Our study found that there were differences in ALFF between the severe OSA group and the control group in many brain regions. In addition, ALFF changes in brain regions were also found after one-night CPAP treatment. Correlation analysis further revealed the relationship between the changes of ALFF in the brain region and the changes of polysomnography parameters. The above results are more obvious in the slow 4-band.

### ALFF differences between the OSA and HC groups

Our study showed that in the slow-5 frequency band, OSA patients showed significantly reduced ALFF in the left inferior temporal gyrus. But in the slow-4 band, the signals of OSA patients were reduced in the bilateral inferior temporal gyrus brain region. The inferior temporal gyrus is related to processing memory and text information, as well as the processing of emotions (28, 29). Previous fMRI studies (8, 30) indicated that the regional cerebral blood flow of the temporal lobe was reduced in OSA patients, and the functional connectivity of the brain regions of the prefrontal, parietal and temporal lobes was reduced in the default mode network (DMN). The results of these studies are consistent with the results of the present study. Therefore, it can be speculated that cognitive dysfunction in severe OSA patients is related to temporal lobe dysfunction.

In addition, OSA patients were found to have signal increased in the right inferior frontal gyrus and left middle frontal gyrus region by analysis of slow-5 and slow-4 frequency bands compared with HC. Previous studies (31, 32) identified that the frontal lobe of OSA patients had a variety of brain function abnormalities. Paul et al. (31) reported that the gray matter volume of the anterior superior frontal gyrus in both hemispheres decreased in OSA patients. The other study (32) reported that children with OSA showed regional homogeneity (ReHo) decreased in the left medial superior frontal gyrus. Sleep fragmentation and hypoxia in OSA affect nocturnal sleep-related recovery processes, causing chemical and structural damage to brain cells. It has recently been reported that sleep disturbances preferentially lead to the prefrontal cortex dysfunction, suggesting that the frontal lobe is a functionally and structurally vulnerable region in OSA patients (31). Although these studies failed to support the changes of ALFF in the frontal lobe, we believe that the increase of ALFF in the frontal lobe may be a compensatory response to hypoxia and sleep fragmentation in severe OSA patients. Previous study (19, 33) reported that the OSA patient group had higher ALFF values in the left medial superior frontal gyrus and right inferior frontal gyrus. These results are consistent with our study. Therefore, long-term chronic sleep deprivation caused by sleep fragments and intermittent hypoxemia in OSA patients may be important factors leading to the dysfunction of the inferior frontal gyrus.

Posterior cingulate gyrus and precuneus are important nodes of the default network. Our study also found that OSA patients showed increased signal in the right posterior cingulate gyrus, right middle cingulate, and left precuneus brain region in the slow-4 band compared with HC. DMN inactivation in OSA patients was found to be abnormally inactivated during working memory tasks and was significantly positively correlated with behavioral performance (34). In addition, the posterior cingulate gyrus is closely related to respiratory control, emotional control and is involved in autonomic functions, including maintaining blood pressure, and salivation. Therefore, the increased cingulate signal in OSA patients may be related to functional compensation. Joo et al. (35) found that OSA reduced gray matter concentration in the cingulate cortex, which could explain clinical manifestations of respiratory, emotional, and cardiovascular disorders. Ayalon et al. (36) found that OSA patients suffered from decreased brain activation in the left precentral gyrus, left anterior cingulate gyrus, and posterior cingulate gyrus during attentional tasks compared with controls, which may also explain the cognitive impairment in OSA patients. Although this study suggested that cingulate gyrus activation was reduced, this study was a fMRI study in the task state, while our study was resting state, which may lead to inconsistent results. In addition, previous studies found that ReHo (37) and ALFF (33) in the cingulate were increased in OSA patients. These signal changes in the cingulate gyrus of OSA patients compared are consistent with our findings. Sleep fragmentation and oxygen reduction may be the key factors in the posterior cingulate gyrus and precuneus dysfunction, which suggests cognitive dysfunction in OSA.

### ALFF differences between the pre-CPAP and post-CPAP

We found that the signal in the left insula increased in the slow-5 frequency band after CPAP treatment. And in the slow-4, the signal increased in the left caudate area, while the signal reduced in the left calcarine area. The human insula is small but the insular cortex has extensive connections to the frontal, temporal, cerebellum, and limbic regions and is involved in a large number of different functions, cortical regulatory regions of emotion and sensorimotor function (38). Zhang's study (39) showed that the ALFF value of the insula was decreased under hypoxia, which may be related to the decreased ventilatory driving force. After one-night CPAP treatment, the blood oxygen saturation improved significantly, so the function of the insula was restored and the signal was enhanced. Therefore, the above study further supported our findings. Qin's study (19) in Tibetans found that the ALFF value of the right insula significantly increased in OSA patients. This study is inconsistent with our results, but the author explained in the article that Tibetans had strong adaptability, that is, they can improve the ventilation function to adapt to hypoxia. Neuroimaging studies suggest that insular dysfunction is thought to be a factor in airway collapse in OSA (40). There is reliable evidence showing that OSA is associated with structural and functional abnormalities of the central nervous system, especially damage in the insular cortex (40). In addition, the insular cortex receives pain and visceral sensory input and has a significant influence on the activity of the sympathetic and parasympathetic nervous systems, and is also involved in the control of certain autonomic functions, including respiration, blood pressure, and salivation (41). The insula regulates sympathetic nerve function, resulting in a decrease in the activity of sympathetic function, leading to hypertension and a high risk of cardiovascular disease in OSA patients. OSA can lead to an increase in blood pressure, but after CPAP treatment, the function of the insular cortex is restored. Therefore, the change of insular function caused by CPAP treatment may be an important mechanism of blood pressure decline in OSA patients after CPAP.

The function of the caudate nucleus is involved in the regulation of respiration. Binks's research (42) suggested that a compensatory increase in blood flow in the caudate nucleus can occur in a hypoxic environment, which is a self-protective response of cerebral blood vessels in a hypoxic environment. We believe that the signal changes of the caudate nucleus after CPAP treatment may be more related to hypoxia, and the improvement of blood oxygen saturation leads to the functional recovery of caudate nucleus. Therefore, after CPAP treatment, blood oxygen saturation increased, caudate nucleus function recovered and its hypoxia compensation was relieved, so the signal increased after CPAP treatment. The above research also supported our findings.

The calcarine cortex change was also found in this article. Only a few studies (43) had reported that OSA patients had significantly longer reaction times than controls in visual tasks, suggesting that these patients have impairments in the underlying mechanisms of visual processing. Future studies can pay more attention to the changes in the function of the calcarine cortex in OSA patients, and whether sleep fragmentation and hypoxia lead to the dysfunction of the calcarine cortex.

Although our study found that ALFF changes in brain regions after one-night CPAP treatment were not consistent with those in OSA patients vs. controls. The possible reason is that one-night CPAP treatment can quickly change the function of brain areas that can be improved after a short period of treatment, and these brain areas may be associated with daytime sleepiness and cognitive function, which can be improved after one-night CPAP treatment. However, the differences of OSA patients and controls are more likely to be related to brain dysfunction caused by long-term chronic effects.

### Correlation analysis

A correlation analysis was performed to identify the relationship between changes in brain function and polysomnography parameters. Our study showed that there was a significant correlation between the left angular and the SWS of sleep. The proportion of SWS in OSA patients after CPAP treatment increased significantly, suggesting that the angular signal was also enhanced after CPAP treatment. The angular gyrus plays an important role in regulating emotion, consciousness, memory and introspection (44). There is also a significant difference between the left angular gyrus and the normal group in the children's OSA study (32). We believe that angular gyrus may be more related to sleep depth. Because children have significantly more deep sleep than adults, it is easier to find differences in children's studies compared to normal people. After CPAP treatment, SWS in OSA patients increased significantly, and there was a significant correlation between SWS and memory improvement. We speculate that the impairment of angular gyrus function in OSA patients may be more related to the reduction of slow wave sleep caused by sleep apnea.

In addition, we found that the cerebellum is associated with sleep architecture and blood oxygen saturation. The slow-5 frequency band was positively correlated with the difference in the REM phase, suggesting sleep rebound in the REM phase more affected the slow-5 frequency band signal in the cerebellum. However, the slow-4 frequency band was related to the mean blood oxygen saturation in the REM and NREM phases, and the correlation between blood oxygen saturation in the REM phase was greater. It suggests that the slow-4 frequency band in the cerebellum is more related to the correction of blood oxygen, especially the blood oxygen saturation in the REM phase. The above results suggested that the slow-5 band in the cerebellar region was related to sleep structure, while the slow-4 band was related to blood oxygen. Therefore, sleep fragmentation and intermittent hypoxia significantly affect the function of cerebellar regions. Studies on brain function in patients with heart failure have shown that cerebellar gray matter loss is associated with hypoxia, which further indicates that hypoxia may lead to cerebellar dysfunction. This research supported our findings (45). Recently, more and more scholars have begun to pay attention to changes in the cerebellum of OSA patients. For example, joo (35) reported that the gray matter concentrations of OSA patients were significantly decreased in biventer lobules in the cerebellum. Park et al. (46) found that OSA showed decreased network integration connectivity in the cerebellum, which was susceptible to hypoxia or ischemia. After long-term CPAP treatment, patients with OSA may have increased cerebellar volume (47, 48). In our study, even one-night CPAP treatment can reverse the brain function of the cerebellum. Therefore, we speculate that effective CPAP therapy can improve oxidative stress in patients with hypoxemia and disturbed sleep architecture, leading to a reversal of cerebellar network integration and cerebellar connectivity. Thus, we conclude that CPAP treatment can reverse cerebellar damage in OSA patients.

Therefore, we believe that one-night CPAP treatment can also find changes in the brain function of OSA, which is similar to changes in brain function after long-term CPAP treatment. We speculate that brain function changes after one-night CPAP treatment may predict the changes after long-term CPAP treatment, and we also can explore the main areas of acute and chronic brain function damage caused by OSA. These injury areas may be related to the complications of OSA patients, such as cognitive dysfunction and hypertension. These corresponding brain regions intervention studies can be carried out in the future to explore the main mechanisms of dysfunction in OSA patients.

There are several limitations in the current study. Only male patients with severe OSA were included in this study, and female and mild-to-moderate OSA patients were not included. The sample size of cases and controls in this study was relatively small.

## Conclusion

We found that the use of the slow-4 frequency band may be more specific in OSA studies. These results suggest that the severe OSA patients have frequency-related abnormal spontaneous neural activity, which may contribute to a better understanding of the pathological basis of OSA-related diseases and may provide potential therapeutic targets for OSA patients.

## Data availability statement

The raw data supporting the conclusions of this article will be made available by the authors, without undue reservation.

## Ethics statement

The studies involving human participants were reviewed and approved by the Human Research Ethics Committee of West China Hospital, Sichuan University. The patients/participants provided their written informed consent to participate in this study.

## Author contributions

YS: conceptualization, data curation, methodology, software, investigation, formal analysis, and writing—original draft. MX: methodology, software, and writing—original draft. SY: methodology and writing—review and editing. KZ: resources, supervision, and writing—review and editing. XT: supervision. All authors contributed to the article and approved the submitted version.

## Funding

This work was supported by the Ministry of Science and Technology of the People's Republic of China (2021ZD0201900) and the National Natural Sciences Foundation of China (82120108002, U21A20335, 81300065, and 8210053444).

## Conflict of interest

The authors declare that the research was conducted in the absence of any commercial or financial relationships that could be construed as a potential conflict of interest.

## Publisher's note

All claims expressed in this article are solely those of the authors and do not necessarily represent those of their affiliated organizations, or those of the publisher, the editors and the reviewers. Any product that may be evaluated in this article, or claim that may be made by its manufacturer, is not guaranteed or endorsed by the publisher.
