# Supplementary material for: Aberrant amplitude of low-frequency fluctuations in different frequency bands and changes after one-night positive airway pressure treatment in severe obstructive sleep apnea
Source: Front Neurol. 2022 Aug 22;13:985321. doi: 10.3389/fneur.2022.985321 (PMC9441702; doi:10.3389/fneur.2022.985321)
Supplement: Supplementary file 1 [file Data_Sheet_1.docx]

# Supplementary Tables1: the relation of whole brain ALFF pre-CPAP and baseline sleep data

| CPAP | Regions | Brodmann area | Cluster Size (Voxels) | Cluster Size (mm^3) | X | Y | Z | Peak Intensity: |
| --- | --- | --- | --- | --- | --- | --- | --- | --- |
| (A) cpap_Slow-5 frequency band (0.01–0.027 Hz)) | | | | | | | | |
| R_AHI_rem | Occipital_Mid_L | BA18_L | 42 | 1134 | -27 | -96 | 18 | 0.657055 |
| R_REM_min | Caudate_L | none | 34 | 918 | -15 | -9 | 27 | -0.69765 |
| R_REM_O2_average | Cerebelum_Crus1_R | BA18_R | 39 | 1053 | 27 | -81 | -18 | -0.74096 |
|  |  |  |  |  |  |  |  |  |
| (B) cpap_(Slow-4 frequency band (0.027–0.073 Hz)) | | | | | | | | |
| R_AHI_rem | Cuneus_L | BA19_L | 42 | 1134 | -3 | -90 | 30 | 0.685723 |
| R_nREM_O2_ average | Vermis_4_5 | BA18_R | 35 | 945 | 3 | -60 | -6 | -0.72202 |
| R_REM_min | Caudate_L |  | 54 | 1458 | -15 | -9 | 24 | -0.69633 |
| R_REM_min | Vermis_9 |  | 57 | 1539 | -3 | -57 | -33 | -0.72432 |
| R_REM_min | Hippocampus_R, | BA35_R | 42 | 1134 | 20 | -10 | -17 | -0.63019 |
| R_REM_min | Lingual_R | BA27_R | 42 | 1134 | 9 | -36 | 0 | -0.63748 |
| R_REM_min | Cerebelum_6_L | BA19_L | 40 | 1080 | -27 | -60 | -21 | -0.66773 |
| R_REM_O2_average | Thalamus_R, |  | 39 | 1053 | 16 | -19 | 20 | -0.64483 |
| R_SWS_min | Angular_L | BA39_L | 47 | 1269 | -39 | -66 | 45 | -0.71904 |

**Note**: All clusters were reported with a voxel-level threshold of P < 0.005, GRF correction, and cluster-level of P < 0.05, two tailed.

R: Relation, REM_min: REM sleep time, AHI_rem: REM stage AHI, nREM_O2_ average: NREM stage mean SaO2, REM_O2_average: REM stage mean SaO2,

# Supplementary Tables2: the relation of whole brain ALFF of post CPAP and baseline sleep data

| PSG | Region | Brodmann area | Cluster Size (Voxels) | Cluster Size (mm^3) | X | Y | Z | Peak Intensity: |
| --- | --- | --- | --- | --- | --- | --- | --- | --- |
| (A) psg_Slow-5 frequency band (0.01–0.027 Hz)) | | | | | | | | |
| R_total_O2_ average | Supp_Motor_Area_R | BA6_R, | 44 | 1188 | 6 | -6 | 78 | -0.66065 |
| (B) psg_(Slow-4 frequency band (0.027–0.073 Hz)) | | | | | | | | |
| R_nREM_O2_ average | Supp_Motor_Area_R, | BA6_R | 49 | 1323 | 0 | 0 | 78 | -0.75478 |
| R_rem_min | Insula_R | BA48_R | 40 | 1080 | 33 | -15 | 15 | -0.65597 |
| R_REM_O2_average | Precuneus_R |  | 36 | 972 | 3 | -63 | 39 | 0.57839 |

**Note**: All clusters were reported with a voxel-level threshold of P < 0.005, GRF correction, and cluster-level of P < 0.05, two tailed.

Note: R: Relation, REM_min: REM sleep time, total_O2_ average: total sleep mean SaO2 I, nREM_O2_ average: NREM stage mean SaO2, REM_O2_average: REM stage mean SaO2,

Whole-brain ALFF before and after CPAP treatment were correlated with sleep-related data, respectively, and regions with significant correlation results were selected as regions of interest (ROIs) and extracted (Supplementary Table1 and Supplementary Table2). Then, we perform the changes between the mean ALFF values of brain regions before and after CPAP treatment in these ROIs and the changes between the sleep data before and after CPAP treatment for correlation analysis.
